# Irisin regulates cardiac physiology in zebrafish

**DOI:** 10.1371/journal.pone.0181461

**Published:** 2017-08-03

**Authors:** Lakshminarasimhan Sundarrajan, Chanel Yeung, Logan Hahn, Lynn P. Weber, Suraj Unniappan

**Affiliations:** Laboratory of Integrative Neuroendocrinology, Department of Veterinary Biomedical Sciences, Western College of Veterinary Medicine, University of Saskatchewan, Saskatoon, Saskatchewan, Canada; University of California, Davis, UNITED STATES

## Abstract

Irisin is a myokine encoded in its precursor fibronectin type III domain containing 5 (FNDC5). It is abundantly expressed in cardiac and skeletal muscle, and is secreted upon the activation of peroxisome proliferator-activated receptor gamma coactivator-1 (PGC-1 alpha). We aimed to study the role of irisin on cardiac function and muscle protein regulation in zebrafish. Western blot analyses detected the presence of irisin protein (23 kDa) in zebrafish heart and skeletal muscle, and irisin immunoreactivity was detected in both tissues. Irisin siRNA treated samples did not show bands corresponding to irisin in zebrafish. In vitro studies found that treatment with irisin (0.1 nM) downregulated the expression of PGC-1 alpha, myostatin a, and b, while upregulating troponin C mRNA expression in zebrafish heart and skeletal muscle. Exogenous irisin (0.1 and 1 ng/g B.W) increased diastolic volume, heart rate and cardiac output, while knockdown of irisin (10 ng/g B.W) showed opposing effects on cardiovascular function. Irisin (1 and 10 ng/g B.W) downregulated PGC-1 alpha, myostatin a and b, and upregulated troponin C and troponin T2D mRNA expression. Meanwhile, knockdown of irisin showed opposing effects on troponin C, troponin T2D and myostatin a and b mRNAs in zebrafish heart and skeletal muscle. Collectively, these results identified muscle proteins as novel targets of irisin, and added irisin to the list of peptide modulators of cardiovascular physiology in zebrafish.

## Introduction

Skeletal muscle constitutes up to 40% of total body weight, and is considered an exercise dependent endocrine organ that constitutes approximately 75% of body proteins [1, 2]. Skeletal muscle regulates cytokines and myokines that exert autocrine and paracrine effects in humans [3–6]. Some of the skeletal muscle derived cytokines, including interleukin-6 have the ability to regulate glucose and lipid levels [7]. Irisin is a recently confirmed, exercise-induced, 23 kDa myokine abundantly expressed in rodent and human skeletal muscle [8]. Irisin is secreted from FNDC5, a 212 amino acid precursor, after the cleavage of its extracellular portion [9, 10]. FNDC5 is regulated by PGC-1 alpha, which forms an integral part of the muscle post-exercise, and causes an increase in energy expenditure in mammals [9]. Processing of FNDC5 by PGC-1 alpha triggers the release of irisin into circulation [9, 11]. FNDC5 mRNA is expressed in the brain, adipose tissue, gut (rectum) and pericardium in humans [12]. Previous results have reported that irisin is present in the cerebrospinal fluid and is expressed in the hypothalamus, adipose tissue and skeletal muscle in humans [13, 14]. Elevated levels of circulating irisin induced expression of thermogenin in white adipose cells, led to browning, and resulted in increased thermogenesis and energy expenditure [15]. Lower expression of FNDC5 has been associated with reduced aerobic performance in humans, contributing to heart failure [15]. Irisin is considered a key promoter in the central nervous system, and it regulates cardiac contractility [16–18]. More recently, irisin has gained importance as a potential biomarker for myocardial infarction due to its abundance in cardiac muscle [9, 19]. Irisin exhibits many biological actions in vertebrates.

Immunohistochemical studies revealed irisin immunopositive cells concentrated in skeletal and cardiac muscles, Purkinje cells in cerebellum and neuroglial cells in rodents [20, 21]. Intracerebroventricular administration of irisin in rats resulted in increased blood pressure and enhanced cardiac contractility [17]. On the other hand, peripheral administration (intraperitoneal injection) of irisin, or irisin injection into the cerebellar area of the nucleus ambiguus decreased blood pressure via vagal stimulation in rats [17, 22]. It has been reported that overexpression of irisin increased energy expenditure, reduced body weight, improved lipid metabolism and glucose tolerance, and suppressed insulin resistance in high fat fed mice [9, 23, 24]. In contrast, knockdown of FNDC5 resulted in a suppressive effect on neural differentiation of embryonic stem cells in mouse [25]. In zebrafish, irisin promotes angiogenesis and modulates matrix metalloproteinase activity through the ERK signaling pathway [26]. Whether irisin exerts any effects on metabolism and cardiac function in non-mammals remains unclear.

Muscle, a major source of irisin, is also a reservoir of other metabolically modulated proteins including PGC-1 alpha, myostatin, troponin and tropomyosin. PGC-1 alpha is an important factor that helps muscle adaptation to endurance exercise [27]. In mice, deletion of PGC-1 alpha resulted in reduced muscle functionality and increased inflammation. Myostatin is member of the transforming growth factor beta family and is a secreted signalling mediator that plays an important role in suppressing the conversion of white adipose tissue to beige/brown adipose tissue in humans [28, 29]. In mice, depletion of myostatin resulted in increased cell mass, decreased body fat deposition, increased insulin sensitivity, increased fat oxidation and protection from obesity [30–32]. In addition, knockout of myostatin increased the expression of AMPK, PGC1 alpha and FNDC5, leading to activation of browning of fat in mice [32]. Myostatin has a negative effect on satellite cell growth and postnatal myogenesis in zebrafish [33]. Troponin, a complex protein consisting of troponin C, troponin I and troponin T, is abundantly expressed in cardiac and skeletal muscle tissues and plays an important role in muscle contraction[34]. Cardiac troponin C is a primary determinant of cardiac contractility since it is a calcium binding protein that directly mediates responses to the amount of intracellular calcium released in the heart[35]. On the other hand, cardiac troponin T is a key mediator protein that binds the troponin complex to tropomyosin to mediate controlled interaction between actin and myosin filaments in the myocardial cells of the heart[36]. Overexpression of troponin T has resulted in myocardial damage and its release into circulation from damaged cardiomyocytes is currently used as a biomarker for diagnosing acute myocardial infarction [37, 38]. It is possible that myostatins and troponins contribute to irisin effects on cardiac and metabolic physiology.

We hypothesized that irisin regulates cardiac function and modulates muscle proteins in zebrafish. The main focus of this research was in determining two important aspects of irisin in zebrafish. First, we elucidated whether irisin has any whole animal effects, by examining cardiac function in zebrafish. Second, we studied irisin effects on the expression of muscle proteins discussed above in zebrafish. The results of this research show a role for irisin in regulating cardiovascular physiology, and identify novel targets of irisin in zebrafish.

## Materials and methods

### Animals

Zebrafish (*Danio rerio*; 2–4 months old; body weight: ~1 g) were purchased from Aquatic Imports (Calgary, Canada) and were maintained at 27°C under 12L:12D photoperiod cycle. All fish were fed once a day up to 3–4% body weight with slow sinking pellets (slow-sinking pellets; Aqueon, Catalog# 06053). Fish were euthanized using 0.5% tricaine methanesulfonate-222 (TMS-222, Syndel Laboratories, BC, Canada) followed by spinal transection. All animal studies complied within the policies of the Canadian Council for Animal Care, and were approved by the University of Saskatchewan Animal Research Ethics Board (2012–0033).

### Western blot analyses

Total protein samples from heart and skeletal muscle was collected to confirm the presence of irisin by Western blot analysis. Fish (n = 6) were euthanized using 0.5% TMS-222 before dissection and tissue collection. Tissues for Western blot were homogenized using T-PER tissue protein extraction buffer (Thermo Scientific, Catalog# 78510) followed by protein concentration determination by Bradford assay using NanoDrop 2000c (Thermo, Vantaa, Finland). The samples were prepared using 1X Laemmli buffer containing 0.2% of 2-mercaptoethanol (Bio-Rad, Catalog# 161–0737 and -0710) and were subjected to boiling at 95°C for 5 min followed by vortexing prior to loading. Tissue total protein samples (40 μL; 15, 30 or 40 μg total protein from heart and skeletal muscle of control zebrafish, or siRNA treated zebrafish), or synthetic irisin (positive control; 10 μg) were loaded and were run on a gradient gel (Bio-Rad, Catalog# 456–1104) at 200V for of 30 min. After the run, proteins were transferred to a 0.2 μm BioTrace nitrocellulose membrane (PALL Life Sciences, Catalog# 27377–000) subjected to blocking using 1X RapidBlock solution (AMRESCO, Catalog# M325). In order to detect the presence of irisin and β-tubulin (reference protein; Catalog # 2146, Cell signalling, Danvers, MA), and rabbit polyclonal FNDC5 antibody (Catalog# ab131390, 1:3000, Abcam, Ramona, Massachusetts) were used. As the secondary antibody, goat anti-rabbit IgG (H+L) HRP conjugate (Catalog# 170–6515, 1:3000, Bio-Rad) was used. For visualization of protein, the membrane was incubated for 5 min in Clarity Western ECL substrate (Bio-Rad, Catalog# 170–5061) and imaged using ChemiDoc MP imaging system (Bio-Rad, Catalog# 170–8280). Membrane stripping for detection of reference protein was conducted using Western blot stripping buffer (Thermo Scientific, Catalog# 46430). Primary antibody was pre-absorbed in 10 μg synthetic human irisin (Catalog# 067–16, Phoenix Pharmaceuticals, Inc, Burlingame, CA) overnight and was used as pre-absorption controls for zebrafish tissues to confirm the specificity of the irisin antibody. Precision plus protein dual Xtra standards (Bio-Rad, Catalog# 161–0377) were used as the marker to detect the molecular weight of irisin and beta-tubulin.

### Immunohistochemistry

The localization of the irisin protein in zebrafish heart and skeletal muscle sections were detected by immunohistochemical (IHC) studies as described in detail earlier [39]. The primary antibody used was rabbit polyclonal FNDC5 antibody (Catalog# ab131390, 1:3000, Abcam, Ramona, Massachusetts) for irisin. The slides were then washed with PBS and then were incubated with secondary antibody for one hour at room temperature. Goat polyclonal anti-rabbit IgG (Catalog# TI-1000, 1:500 dilution, Vector Laboratories, California) was used as secondary antibody for irisin respectively. The slides were then rewashed with PBS and were mounted on Vectashield medium containing DAPI dye (Blue, Vector Laboratories). The slides were dried, and imaged using a Nikon inverted microscope (L100) (Nikon DS-Qi1 MC camera, ON, Canada) and analyzed using NiS Elements imaging software (Nikon, Canada). For controls, primary antibody was pre-absorbed in 10 μg synthetic human irisin (Catalog# 067–16, Phoenix Pharmaceuticals, Inc, Burlingame, CA) overnight to confirm the specificity of the irisin antibody in zebrafish tissues. Tissue sections incubated with secondary antibody alone, or preabsorbed using synthetic irisin, were used as negative controls.

### Tissue culture studies

Samples of heart and skeletal muscle from zebrafish (n = 8/group) were collected upon euthanasia using 0.5% tricaine methanesulfonate (TMS-222, Syndel Laboratories, BC, Canada). Tissues were added to each plate and were incubated in the fresh media for a period of 2 hours. The media for tissue culture had the following components; DMEM 1X (500 ml); NAHCO3 (1.85g); pencillin/streptomicin (5.5 ml) and gentamicin (250 mg). Different concentrations of irisin (0, 0.1 and 10 nM) were prepared containing the fresh media were freshly prepared during this incubation period. Post incubation, plates were replaced with media containing irisin at different concentrations. The plates were incubated for 60 or 120 mins respectively. The tissues were then collected and stored at -80°C until further analysis. In order to determine the effect of irisin on muscle proteins, tissues collected were used for studying the relative mRNA expression of troponin C, PGC-1 alpha, myostatin-a and myostatin-b and normalized to 18s RNA (housekeeping gene) (Table 1). qPCR was carried out using iQ SYBR Green supermix (Bio-Rad, Catalog#170–8880) and CFX Connect Optics module system (BioRad, Canada) controlled by CFX Connect PC-based software (BioRad, Canada).

**Table 1:** Forward and reverse primers, and the annealing temperature used in PCR and qRT-PCR analyses of the expression of mRNAs of interest.

Gene	Primer sequence (5’-3’)		Annealing temperature (°C)
	Forward	Reverse	
FNDC5b	GCTTATATCTTCGCGTCCTC	GCCAGTTTCTCTGACTCTTT	59
PGC- 1 alpha	TCTATTCGGAAGGGCCCAGA	GGTGGTGCTGTCTCGTTTTG	58
Myostatin-b	TCCTTTAGCACGCCTTGGAA	TGCTTGAGTCGGAGTTTGCT	60
Myostatin-a	TTTTGAGCATCCTGCGCCTA	ATCTTTGGGCTCAGTGCGAA	60
Troponin-C	GCAGAAAAATGAGTTCCGTGC	TTCCGCCAGTTCTTCCTCTG	60
Troponin T2D	AGTTCAGGAGGAAGTGGATGAGT	AGTCTGGCTTGACGCTCTTTC	60
β-Actin	CTACTGGTATTGTGATGGACT	TCCAGACAGAGTATTTGCGCT	59
18s	GGATGCCCTTAACTGGGTGT	CTAGCGGCGCAATACGAATG	60

### Dose dependent effects of irisin on cardiac function in zebrafish

Cardiac function was assessed in zebrafish using a VEVO 3100 high frequency ultrasound machine (Visualsonics, Markham, ON), using B-mode imaging as described earlier [39, 40]. Zebrafish (4 months old) were injected intraperitoneally (6 μL) with synthetic human irisin (Catalog# 067–16; Phoenix pharmaceuticals, Burlingame, CA) at doses 0.1, 1, 10 ng/g body weight. The control group were injected with 6 μL of saline (0.9% sodium chloride). The siRNA sequences of irisin and a scrambled control siRNA are provided in Table 2. Zebrafish irisin siRNA was synthesized by Dharmacon (Montreal, CA). The control group (n = 6) were injected with saline (0.9% sodium chloride; Baxter corporation, Catalog# JB1323) (Table 2). Scrambled irisin siRNA was designed to test whether any siRNA sequence that shares same set of mRNAs and length but was highly dissimilar in the arrangement could elicit same biological effect on zebrafish. Scrambled siRNA sequence was designed using Genscript sequence scramble tool (https://www.genscript.com/ssl-bin/app/scramble) from the corresponding zebrafish siRNA sequence and synthesized by Dharmacon (Montreal, CA) (Table 2). Zebrafish irisin siRNA and scrambled siRNA were injected intraperitoneally at 10 ng/g B.W. Each fish was allowed to recover for a period of 15 minutes. Zebrafish were anesthetized prior to ultrasound experiments using 20 mg/L Aquacalm (Syndel Laboratories, Canada). Fish were then transferred to a groove in a styrofoam-lined holding dish and placed ventral side up, with aerated, 27±0.5°C water containing 20 mg/L Aquacalm superfusing the fish to maintain anesthesia throughout ultrasound testing and minimal impact towards cardiovascular function in zebrafish [39, 40].

**Table 2:** Forward and reverse sequences of irisin and scrambled siRNAs.

siRNA	siRNA sequence (5’-3’)		Length
	Forward	Reverse	
**Irisin**	C.C.A.A.A.G.A.G.U.C.A.G.A.G.A.A.A.C.U.U.U	P.A.G.U.U.U.C.U.C.U.G.A.C.U.C.U.U.U.G.G.U.U	21
**Scrambled**	G.C.G.U.A.U.C.A.A.C.G.G.A.G.U.U.A.U.A.U.U	P.U.A.U.A.A.C.U.C.C.G.U.U.G.A.U.A.C.G.C.U.U	21

A MX700 scan head was used to obtain short and long-axis views of the zebrafish ventricle in B-mode. The areas of three different short axis views along the ventricle were measured as A1, A2, A3 while the ventricular length of long axis view was measured and divided by three to give ventricular height (h) as per Eq (1). All of these values were measured at both systole and diastole volume using Visualsonics software (Markham, ON). Using these values, end systolic and diastolic volumes (mm^3^ = μl) were calculated for each zebrafish ventricle using the equation:

V=(A1+A2) h+((A3h)/2)+(π/6(h3)) (1)

Stroke volume (SV) was obtained by subtracting end systolic volume from end diastolic volume. Heart rate measurements were calculated by counting the number of heart beats per 10 s during the B-mode ultrasound video loop and converted to beats per minutes (bpm). Cardiac output was measured by multiplying heart rate and stroke volume (SV):

Cardiac output=bpm * SV (2)

The body weight of the fish was measured and all cardiac volume and output data were normalized to body weight and statistical analysis was conducted.

### Regulation of muscle proteins post irisin injection

Zebrafish (n = 8/group; 4 months old) were maintained as described earlier. On the day of the study, synthetic human irisin peptide was intraperitoneally injected (6 μL) at different doses [0, 0.1,1 and 10 ng/g body weight (BW)]. Zebrafish euthanasia, tissue collection and processing were conducted as described earlier. Tissues collected were used for studying the expression of muscular proteins such as troponin C, PGC- 1 alpha, beta-actin, myostatin-a and myostatin-b. Data were normalized to 18s RNA (housekeeping gene) (Table 1).

### Effect of irisin siRNA on muscle proteins in zebrafish

Zebrafish (n = 6/group; 4 months) were maintained as described earlier. On the day of the experiment, irisin siRNA and scrambled irisin siRNA were intraperitoneally injected at 0 and 10 ng/g B.W (Table 2). One hour post-injection, zebrafish were euthanized using 0.5% TMS, spinal transected, and heart and muscle tissues were collected, and stored at –80°C for total RNA extraction. In order to determine the effect of exogenous irisin siRNA on muscle proteins, RT-qPCR were carried out for troponin C, PGC-1 alpha, beta-actin, myostatin-a and myostatin-b. Data were normalized to 18s RNA (housekeeping gene) (Table 1).

### Statistical analysis

Data were analyzed using one-way ANOVA followed by Tukey’s multiple comparison test, post hoc or Tukey Kramer's (t-test). PRISM version 5 (GraphPad Inc., USA) and IBM SPSS version 21 (IBM, USA) were used for statistical analysis. *P* < 0.05 was considered statistically significant. Data are represented as mean + SEM. For ultrasound data analysis, data were normalized to the body weight of fish, and one-way analysis of variance (ANOVA) followed by Fisher’s post hoc test was conducted.

## Results

### Irisin was detected in heart and muscle tissues of zebrafish

**Fig 1:**
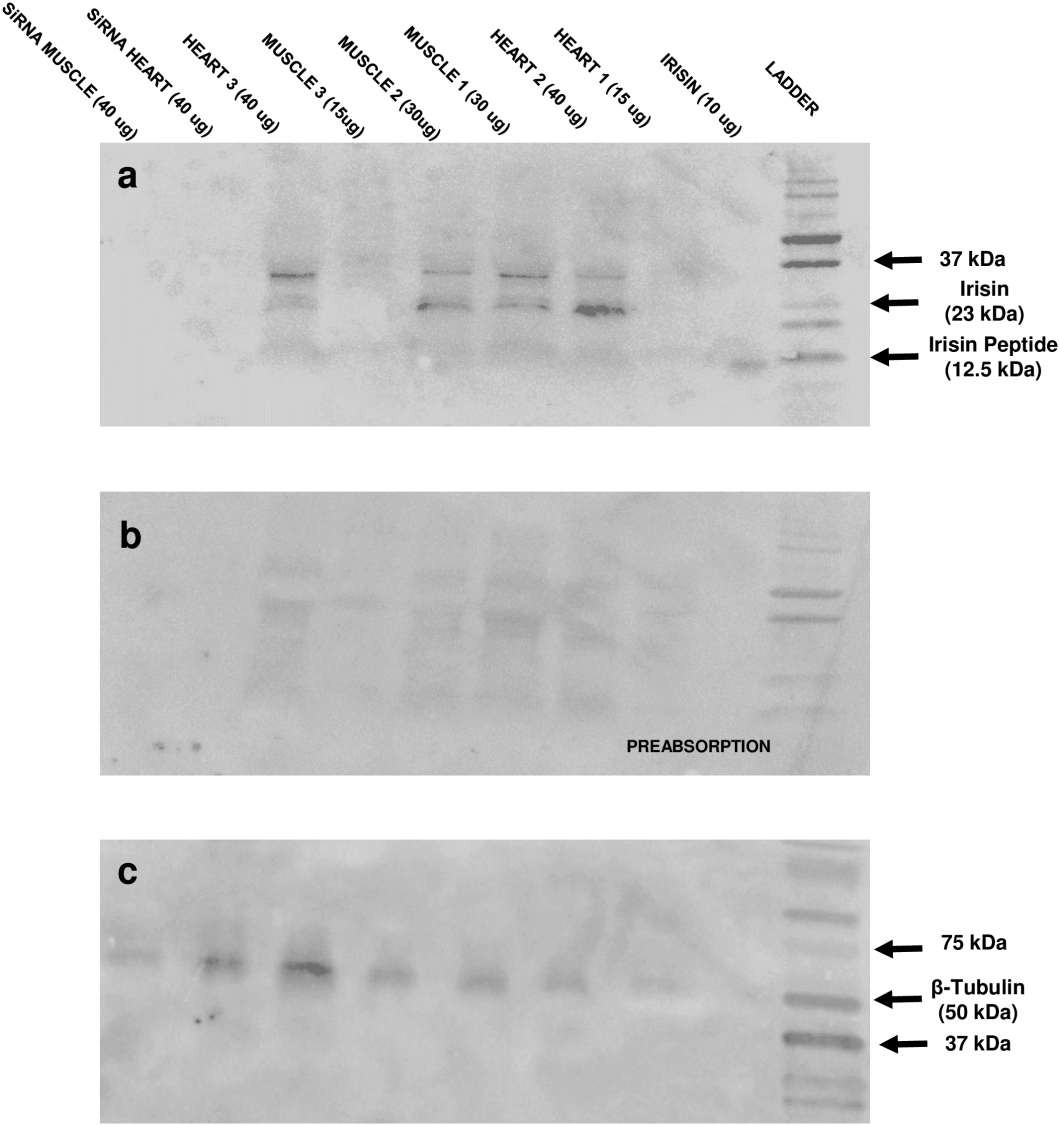
Irisin was detected in the heart and skeletal muscle of zebrafish by Western blot analyses. (**A**) Western blot showing irisin in different concentrations of total protein extract from heart and skeletal muscle of untreated zebrafish (tissue name, and concentration of protein loaded in brackets), and siRNA treated zebrafish (labeled siRNA-heart; siRNA-muscle), and synthetic irisin (positive control); (**B**) pre-absorption control using total protein as indicated in A (age: 2 months; n = 6 zebrafish). Fig (**C**) shows the internal control (β-tubulin) expression in the above tissues. Western blot detected irisin protein at 23 kDa (**A**) while no protein was detected in the preabsorption control (**B**) in skeletal muscle and heart tissues of zebrafish. A band representing beta tubulin was detected in all tissue samples tested.

Western blot analysis detected irisin protein at 23 kDa in zebrafish (2 months old) heart and skeletal muscle (Fig 1A). No bands were detected in tissues from zebrafish treated with irisin siRNA (Fig 1A). Preabsorbed samples using synthetic irisin did not detect any band in zebrafish tissues (Fig 1B). β-tubulin was used as the reference protein (Fig 1C). Beta tubulin was visible in all samples (Fig 1C).

### Irisin immunoreactivity was detected in skeletal and cardiac muscle of zebrafish

**Fig 2:**
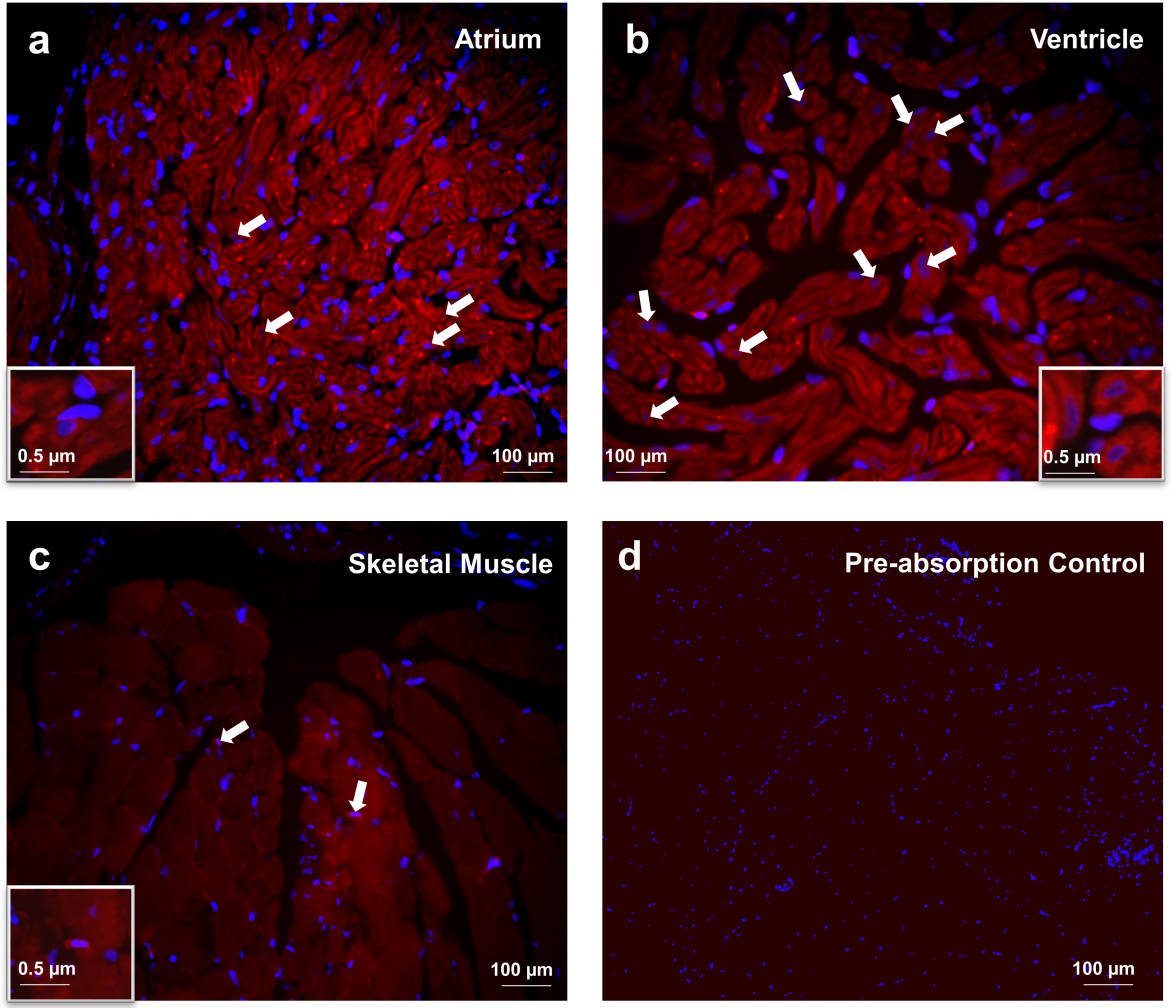
Irisin immunoreactivity was detected in the atrium (A) and ventricle (B) of heart and (C) skeletal muscle tissues of zebrafish. Irisin immunoreactivity was detected in atrial and ventricular cardiomyocytes of zebrafish **(A,B)** In skeletal muscle, irisin immunoreactivity (Catalog# ab131390, 1:3000, Abcam, Ramona, Massachusetts) was detected at the myofibril filament within the myotubule **(C)**. Preabsorption control of irisin was used as negative control **(D)**. Nuclei are stained blue (DAPI). Images were taken at 40X magnification and scale bar = 100 μm (and 0.5 μm for inset).

Irisin immunoreactivity (red) was detected in the atrial and the ventricular cardiomyocytes of zebrafish (Fig 2A and 2B). Irisin immunoreactivity was also detected in zebrafish skeletal muscle (Fig 2C). DAPI (blue) stained the nuclei of cells (Fig 2A–2D). No immunoreactivity was observed in sections stained with secondary antibody alone, and in preabsorption controls (Fig 2D).

### Irisin downregulates PGC-1 alpha, myostatin a and myostatin b mRNA expression, and upregulates troponin C mRNA expression *in vitro*

**Fig 3:**
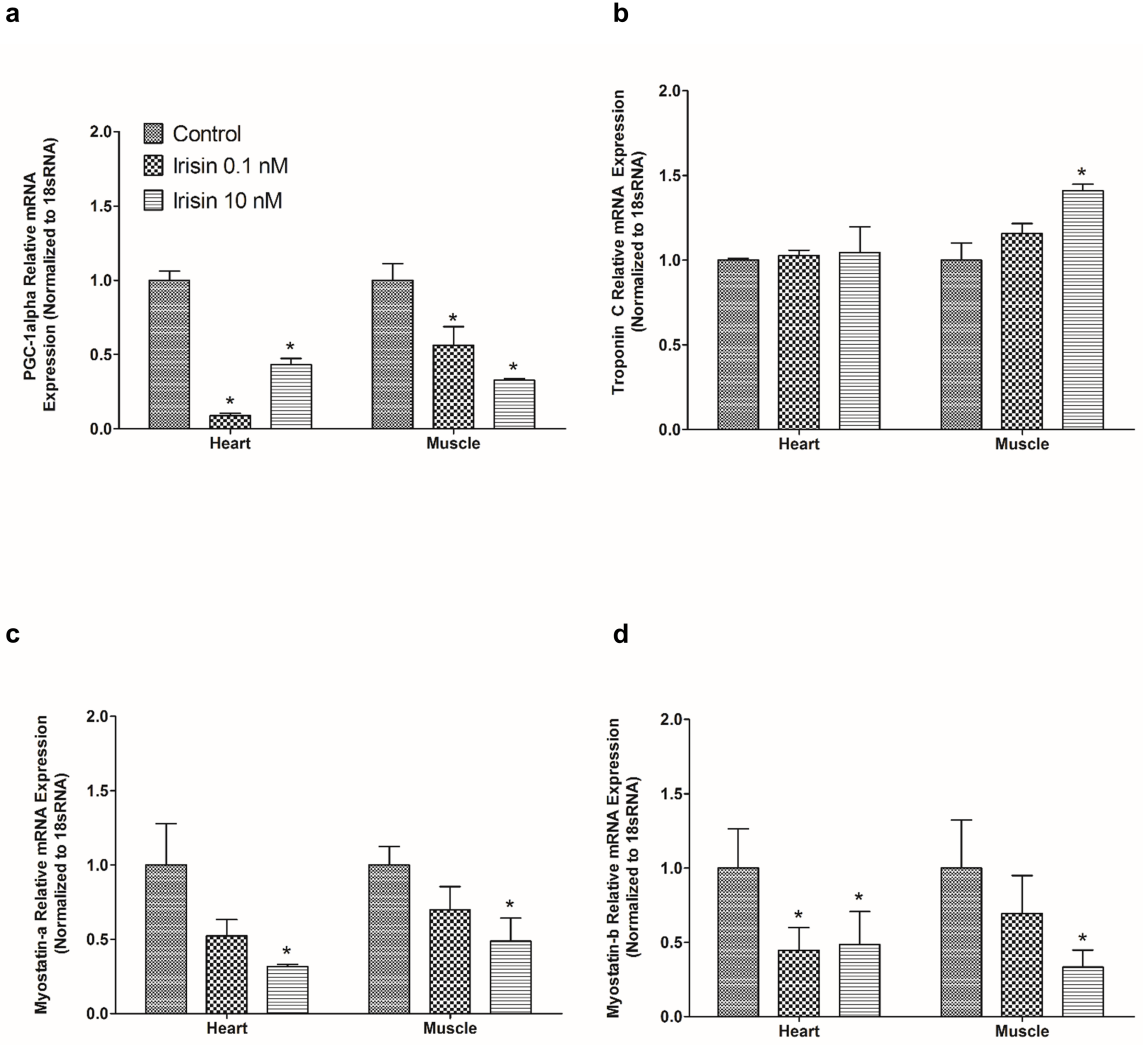
Irisin downregulated mRNA expression of PGC-1 alpha, myostatin-a and b and upregulated troponin C mRNA expression in zebrafish. *In vitro* studies showed irisin (0.1 nM, 10 nM) downregulated relative mRNA expression of PGC- 1 alpha **(A)** in zebrafish heart (n = 8/ group). However, irisin (10 nM) upregulated troponin C relative mRNA expression in zebrafish skeletal muscle **(B).** Irisin (10nM) downregulated myostatin-a relative mRNA expression in zebrafish heart and skeletal muscle **(B)**. Irisin (10 nM) downregulated the relative mRNA expression of myostatin-a in zebrafish heart and muscle **(C)**. Irisin (0.1 nM, 10 nM) downregulated myostatin-b relative mRNA expression when compared to control in zebrafish heart **(D)**. The mRNA expression data was normalized to 18s RNA. Asterisks denote significant difference between the tissues (* p<0.05). Data are presented as mean + SEM. One-way ANOVA (non-parametric) followed by Tukey’s multiple comparison test was used for statistical analysis.

Irisin (0.1 nM, 10 nM) downregulated relative mRNA expression of PGC-1 alpha (Fig 3A) when compared to saline treated controls in zebrafish heart. In contrast, irisin (10 nM) upregulated relative mRNA expression of troponin C in zebrafish skeletal muscle (Fig 3B), while irisin (10 nM) downregulated the relative mRNA expression of myostatin-a in zebrafish heart and muscle (Fig 3C). No significant effect on troponin C relative mRNA expression was detected in zebrafish heart at 0.1 nM, and 10 nM, when compared to controls (Fig 3B). Irisin (0.1 nM, 10 nM) downregulated relative mRNA expression of myostatin b (Fig 3D) when compared to controls in zebrafish heart.

### Irisin modulates cardiac function in zebrafish

**Fig 4:**
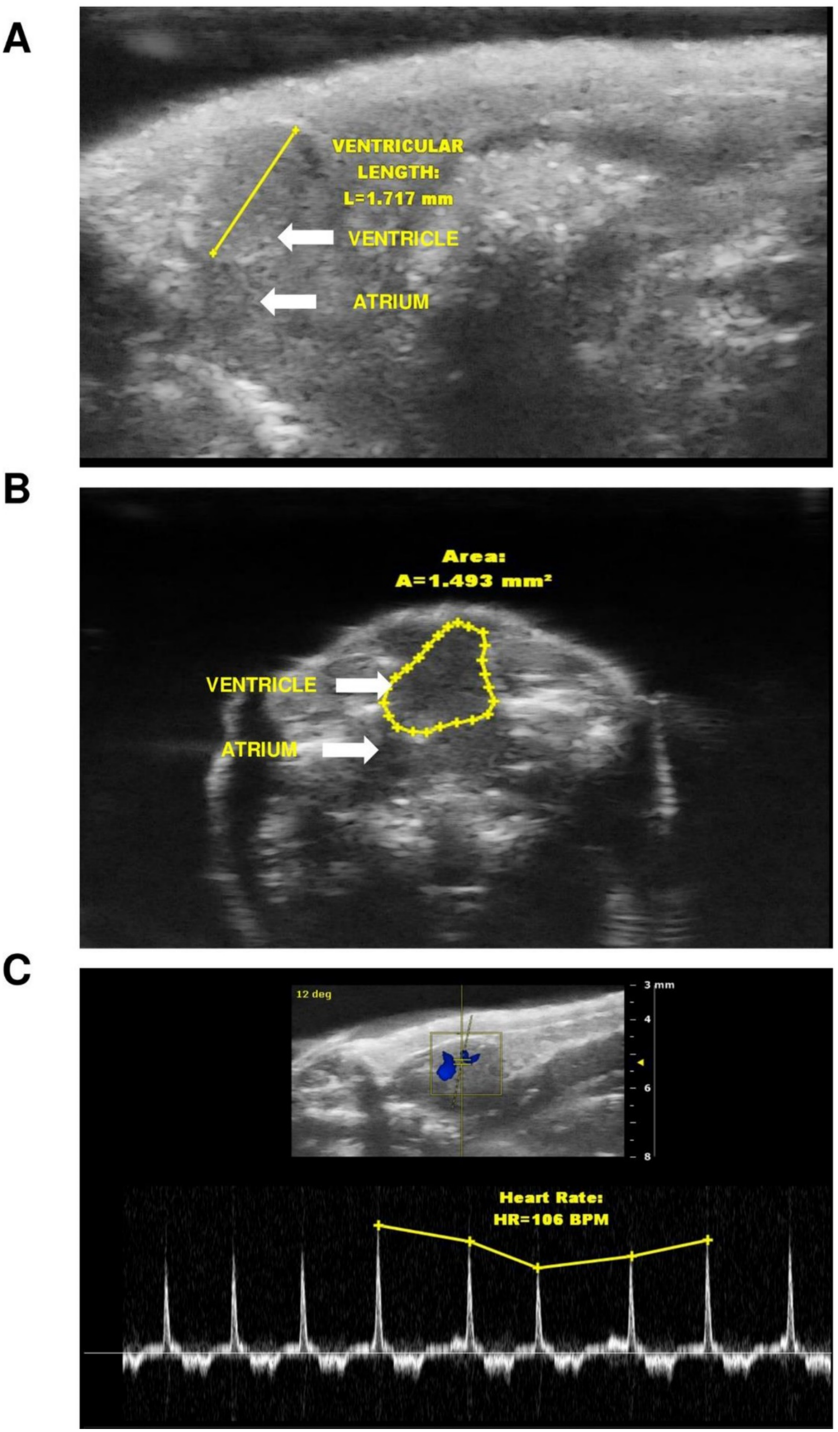
Representative long-axis (A), short axis (B) brightness mode (B-mode) view of adult zebrafish heart. Zebrafish were anesthetized, imaged and the long axis mode was considered to calculate the ventricular length **(A)**, while short axis was considered to calculate ventricular area **(B)** (represented by blue line). Blood flow from atrium to ventricle through the atrioventricular valve are indicated by color (blue) in panel C using color flow Doppler mode and heart rate measurements were calculated by number of heart beats per 10 s during the B-mode ultrasound video loop, and converted to beats per minutes (bpm).

**Fig 5:**
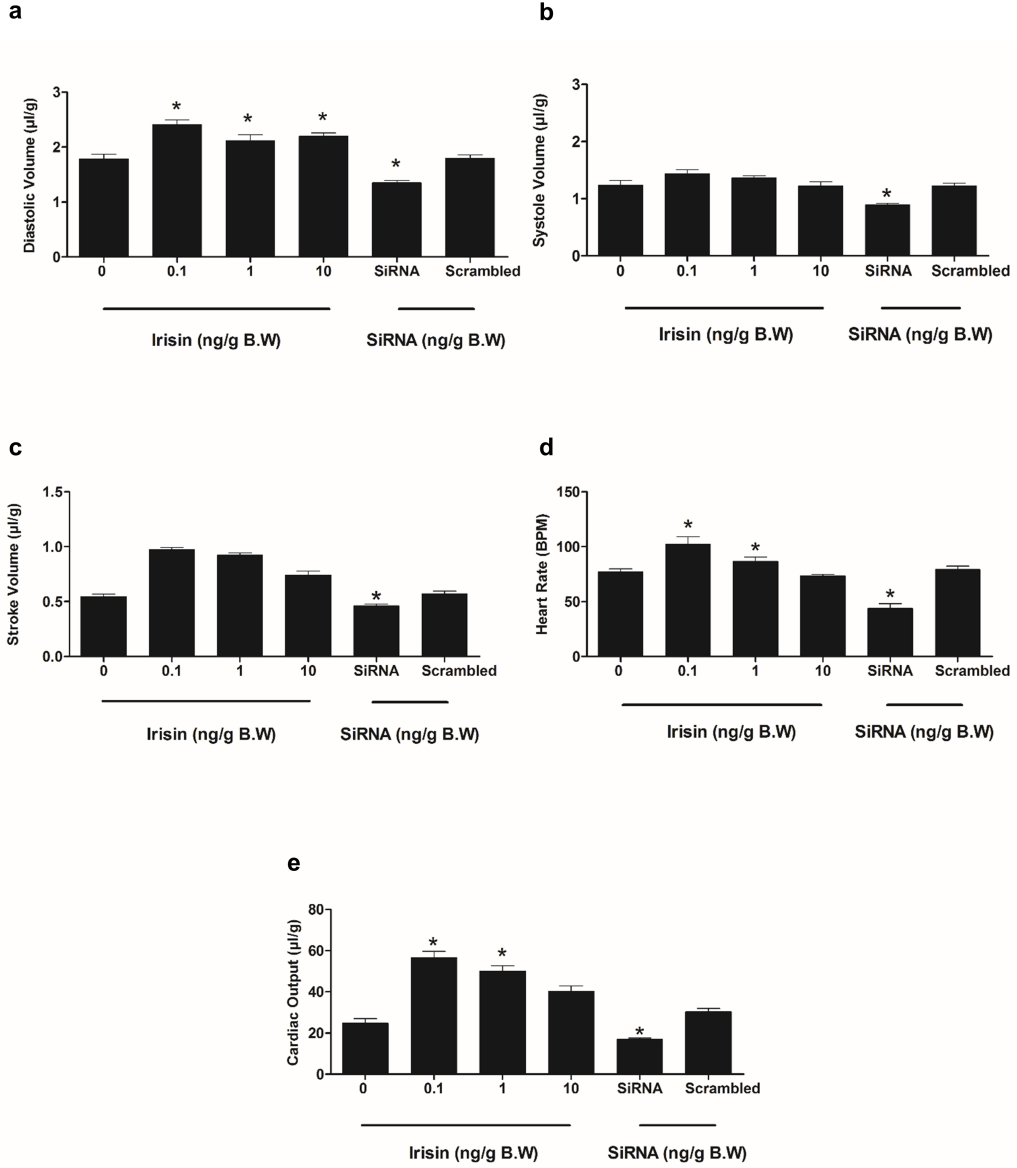
Irisin administration enhances cardiovascular function, while knockdown of irisin by siRNA attenuated cardiac physiology in zebrafish. Exogenous irisin (0.1 ng/g, 1 ng/g and 10 ng/g B.W) increased diastole volume in zebrafish **(A).** siRNA knockdown of irisin (10 ng/g B.W) significantly decreased diastolic volume, systolic volume, stroke volume, heart rate and cardiac output in zebrafish **(A-E).** Exogenous irisin (0.1 ng/g and 1 ng/g) improved heart rate and cardiac output **(D, E)** in zebrafish. No significant effects on cardiovascular functions were observed upon injection of irisin scrambled siRNA, when compared to control group **(A-E)**. Asterisks denote significant differences between control (saline group) and irisin, siRNA injected groups (* p<0.05, n = 6 fish/group). Data are represented as mean + SEM. One-way ANOVA (non-parametric) followed by Tukey’s multiple comparison test were used for statistical analysis.

Representative long-axis brightness mode (B-mode) and color flow Doppler short axis views from the ultrasonography of adult zebrafish heart are shown in Fig 4A–4C. A single intraperitoneal injection of irisin (0.1 ng/g, 1 ng/g and 10 ng/g B.W) increased end-diastolic volume in zebrafish (Fig 5A). Irisin (0.1 and 1 ng/g B.W) also increased heart rate and cardiac output in zebrafish (Fig 5D and 5E). siRNA enabled knockdown of irisin (10 ng/g B.W) significantly decreased end-diastolic volume, end-systolic volume, stroke volume, heart rate and cardiac output in zebrafish (Fig 5A–5E). No significant effect on cardiac function was observed in response to 10 ng/g B.W irisin scrambled siRNA injection, when compared to control group (Fig 5A–5E).

### Irisin downregulates PGC-1 alpha, myostatin a and b, and upregulates troponin C and troponin T2D *In vivo* in zebrafish

**Fig 6:**
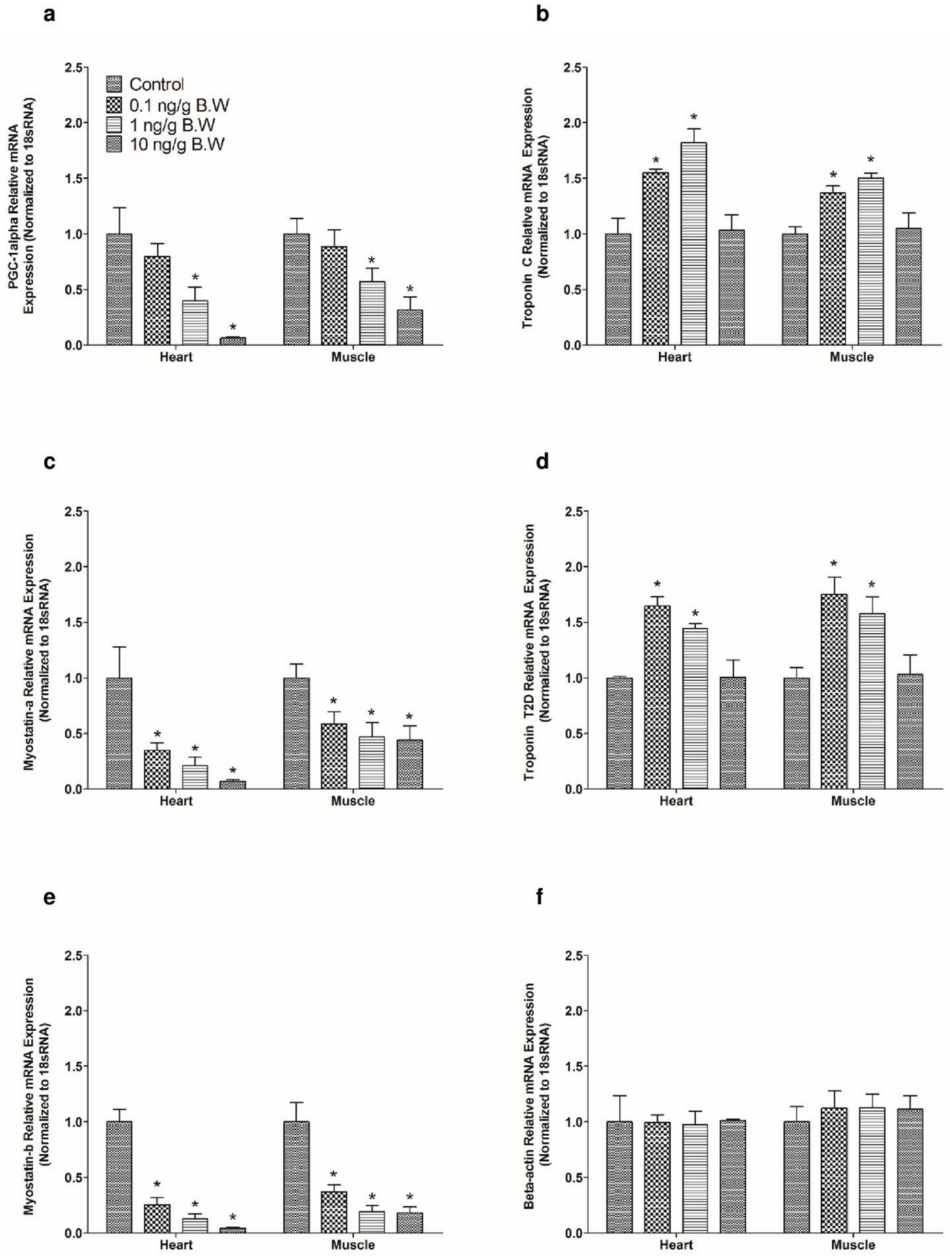
Exogenous irisin administration downregulated PGC-1 alpha, myostatin-a and b mRNA expression and upregulated troponin C and T2D mRNA expression in zebrafish. Exogenous irisin (1 ng/g and 10 ng/g B.W) significantly downregulated PGC-1 alpha **(A)** relative mRNA expression in zebrafish heart and skeletal Irisin (0.1 and 1 ng/g B.W) upregulated troponin C mRNA expression **(B)** in zebrafish heart and skeletal muscle when compared controls. However, exogenous irisin (0.1 ng/g 1 ng/g and 10 ng/g B.W) downregulated myostatin-a relative mRNA expression in zebrafish muscle and heart **(C)**. Upon exogenous administration of irisin at 0.1 ng/g and 1 ng/g B.W, troponin T2D relative mRNA expression was downregulated in zebrafish heart and skeletal muscle **(D)**. Also, irisin (0.1 ng/g, 1 ng/g and 10 ng/g B.W) downregulated myostatin-b relative mRNA expression in zebrafish when compared to saline treated controls **(E)**. No significant effect was observed in response to i.p injection of irisin towards beta-actin relative mRNA expression in heart and muscle of zebrafish **(F)**. Asterisks denote significant differences between control (saline group) and irisin, siRNA injected groups of the same (* p<0.05, n = 8 fish/group). Data are represented as mean + SEM. One-way ANOVA followed by Tukey’s multiple comparison post hoc test were used for statistical analysis.

Exogenous administration of irisin (1 and 10 ng/g B.W) downregulated PGC-1 alpha mRNA expression (Fig 6A) in zebrafish heart and muscle when compared to saline treated controls. On the other hand, exogenous irisin (0.1 and 1 ng/g B.W) upregulated troponin C (Fig 6B) relative mRNA expression in zebrafish heart and skeletal muscle. Intraperitoneal administration of 0.1 ng/g B.W, 1 ng/g B.W and 10 ng/g B.W downregulated myostatin a (Fig 6C) relative mRNA expression in zebrafish heart and skeletal muscle when compared to saline treated group. Also, exogenous irisin (0.1 and 1 ng/g B.W) upregulated troponin T2D (Fig 6D) relative mRNA expression in zebrafish heart and skeletal muscle. Exogenous administration of irisin at 0.1 ng/g B.W, 1 ng/g B.W and 10 ng/g B.W downregulated myostatin b (Fig 6E) relative mRNA expression in zebrafish heart and skeletal muscle when compared to saline treated group. No significant effect was observed in response to an intraperitoneal injection of irisin (0.1 ng/g, 1 ng/g and 10 ng/g B.W) on beta-actin relative mRNA expression in zebrafish heart and skeletal muscle (Fig 6F). Also, no effect in PGC-1 alpha (Fig 6A) mRNA expression was observed after administration of 0.1 ng/g B.W of irisin in zebrafish heart and skeletal muscle.

### Knockdown of irisin upregulates myostatin a and b, and downregulates PGC-1 alpha, troponin C and troponin T2D in zebrafish

**Fig 7:**
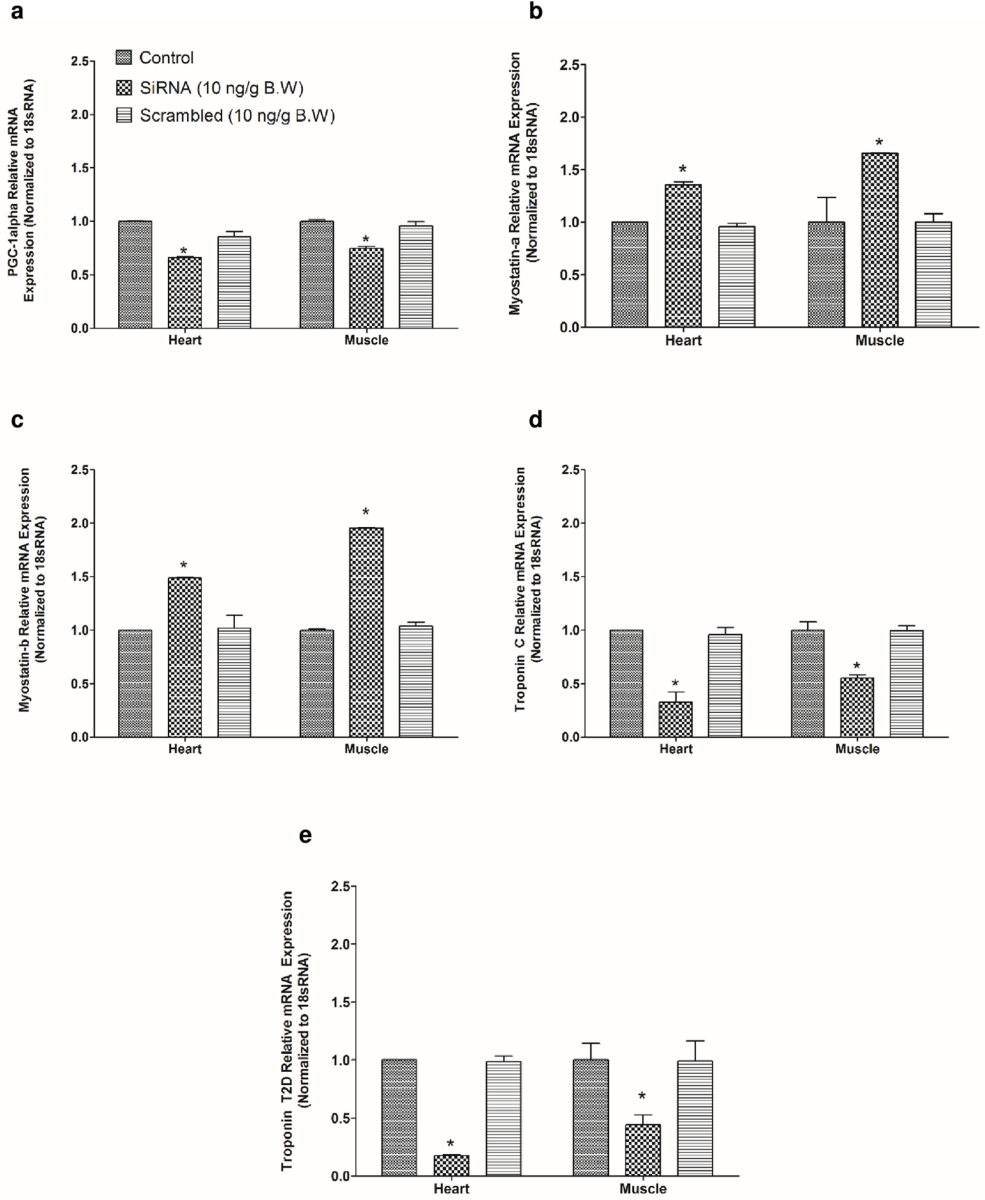
Knockdown of irisin by siRNA altered the expression of mRNAs encoding cardiac muscle proteins in zebrafish. *In vivo* administration of irisin siRNA (10 ng/g B.W) downregulated PGC- 1 alpha **(A)** mRNA expression in zebrafish heart and skeletal muscle samples when compared to control group. However, irisin siRNA (10 ng/g B.W) significantly upregulated myostatin a (**B**) and myostatin b (**C**) relative mRNA expression in heart and muscle tissues of zebrafish when compared to control group **(B, C)**. In addition to that, knockdown of irisin by siRNA downregulated troponin C and troponin T2D relative mRNA expression in zebrafish heart and skeletal muscle when compared to saline treated controls **(D, E)**. No significant effect on PGC- 1 alpha, troponin C, troponin T2D, myostatin a and b relative mRNA expression was observed when scrambled siRNA was injected **(A-E)**.

Zebrafish irisin siRNA (10 ng/g B.W) downregulated PGC- 1 alpha (Fig 7A) mRNA expression in zebrafish heart and muscle. *In vivo* (intraperitoneal) administration of zebrafish irisin siRNA (10 ng/g B.W) upregulated myostatin a and b (Fig 7B and 7C) relative mRNA expression in zebrafish heart and skeletal muscle, when compared to saline treated control group. Irisin siRNA downregulated troponin C (Fig 7D) and troponin T2D (Fig 7E) relative mRNA expression in heart and skeletal muscle, when compared to saline treated control group. Injection of scrambled irisin siRNA (10 ng/g B.W) did not elicit any effects on myostatin a and b, PGC-1 alpha, troponin C and troponin T2D relative mRNA expression in zebrafish tissues (Fig 7A–7E).

## Discussion

Irisin is a myokine derived from skeletal muscle in response to exercise, which has been shown to play a role in regulating cardiac function and angiogenesis in mice [9, 41]. Circulating irisin and adipose tissue FNDC5 were found to attenuate hyperglycemia, visceral adiposity and extramyocellular lipid deposition in obesity and type 2 diabetes in humans [42, 43]. Lower levels of irisin was detected in circulation of individuals with gestational diabetes mellitus [44]. These results suggest a role for irisin in the maintenance of energy homeostasis in normal and disease conditions. In this research, we focused on cardiac expression of irisin and its role in cardiac physiology and muscle proteins in zebrafish. Irisin immunoreactivity was detected in zebrafish atrial and ventricular cardiomyocytes. Irisin immunoreactivity was detected in the myofibrils within the myotubules in skeletal muscle of zebrafish. These observations are in agreement with previous results that detected irisin immunoreactivity in the perimysium, endomysium and skeletal muscle nuclei in Sprague Dawley rats, with a reported increase in cell specific expression upon exercise [45]. In addition to that, abundant irisin immunoreactivity was detected in the myocardium and connective tissues of heart in young rats. In the current study, the cell specific localization of irisin in skeletal and heart muscle tissues suggests that irisin is a key promoter in regulating muscle proteins and cardiac function in zebrafish.

Our next study focused on determining whether irisin has any effects on cardiac physiology and modulation of muscle proteins in zebrafish. *In vivo* administration (i.p.) of irisin elicited an overall stimulatory effect on cardiac function in zebrafish. Intracerebroventricular injection of irisin increased cardiac output and blood pressure in rats by activating hypothalamic paraventricular nuclei (PVN) neurons, while peripheral (intravenous) administration, or injection into the nucleus ambiguus decreased blood pressure, decreased cardiac function and caused vasodilation [17, 22]. A limitation in comparing these studies lies in the routes of administration (central *vs*. intravenous *vs*. intraperitoneal) chosen. The discrepancies in outcomes obtained in these studies could be due to variations in routes of administration, doses used, species-specificity, and mechanism of action in model organisms tested. Cardiac stimulatory effects of irisin are, at least in part, mediated by β-adrenoceptors and irisin-mediated sympathetic stimulation, based on competitive effects with atenolol in rats [17]. However, vagal stimulation was instead suggested to mediate the previously reported decrease in cardiac function and blood pressure reported in rats injected with irisin in the nucleus ambiguous [22]. Overall, at the doses tested, using the i.p route of administration, irisin plays a stimulatory role on cardiac filling, contractility and heart rate in zebrafish. Some of these effects were found at the lower dose(s) tested, while at higher concentrations the responses were diminished or disappeared. One possibility is that at the higher doses of irisin tested, there might be receptor desensitization or downregulation, which results in the lack of responses found at lower doses. The mechanisms and pathways that mediate *in vivo* irisin actions on zebrafish cardiovascular biology warrant further studies.

In an attempt to understand possible mechanisms of irisin action, we then measured muscle proteins that were found to change in response to irisin in our *in vitro* studies. Our *in vitro* studies revealed that irisin (0.1 nM and 10 nM) downregulated PGC-1 alpha mRNA expression in zebrafish heart and muscle. Similarly, exogenous administration of irisin (1 ng/g and 10 ng/g B.W) reduced PGC- 1 alpha, and myostatin a and b relative mRNA expression in zebrafish. This reduction in the expression of PGC-1 alpha relative mRNA expression is in accordance with previous studies that found irisin secretion from skeletal muscles upon PGC-1 alpha activation during endurance training [9]. Knockdown of irisin upregulated myostatin a and b mRNA expression in zebrafish heart and skeletal muscle tissues. Previous results have reported that increased expression of myostatin led to muscle wasting during aging in humans [46]. Overexpression of myostatin resulted in immobilization leading to muscle atrophy in mice [47]. Whether the overexpression of myostatin a and b could influence muscular development and muscle physiology in zebrafish needs further investigation. Administration of irisin resulted in a significant increase in troponin C and troponin T2D mRNA in zebrafish skeletal muscle and heart, while knockdown of irisin downregulated troponin T2D and troponin C in heart and skeletal muscle tissues of zebrafish. Troponin T2D plays an important role in sarcomere assembly and regulation of actin-myosin activity in zebrafish muscle tissues [35, 48]. Morpholino knockdown of troponin T in zebrafish caused suppressive effect in pre-myofibril production leading to abnormalities in myofibrillogenesis [49]. Abnormality in troponin T expression was responsible for uncoupling myofibrillar calcium sensitivity in humans with hypertrophic obstructive cardiomyopathy [50]. Collectively, these results identify myostatin and troponin as novel targets of irisin. Further research to elucidate the mediators and mechanism of cardiac action of irisin is warranted.

## Conclusion

**Fig 8:**
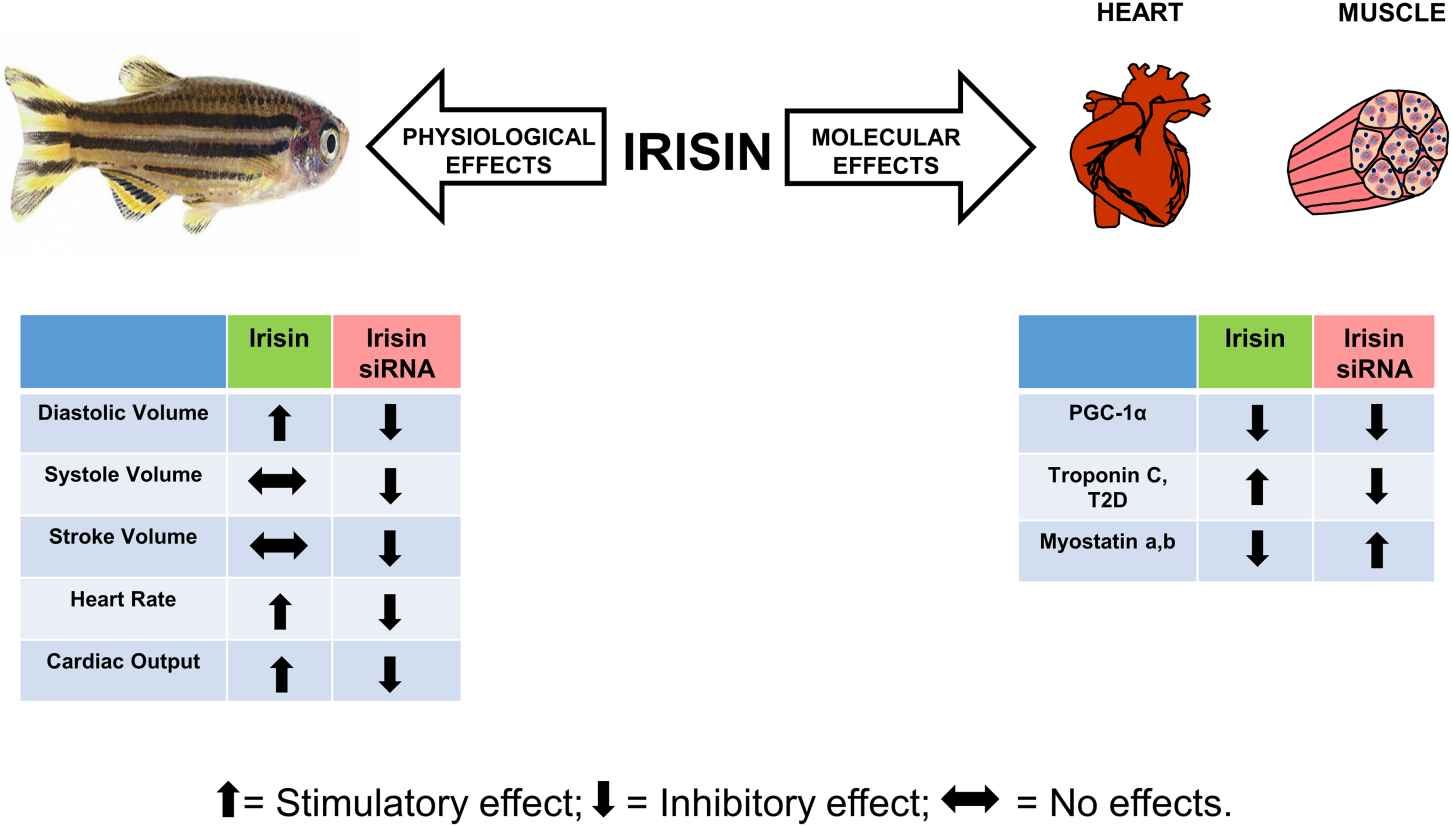
Summary of irisin effect on cardiac function and regulation of muscular proteins in zebrafish. Scheme depicting the role of irisin on cardiovascular physiology, and in the regulation of muscle proteins in zebrafish. Irisin is abundantly expressed in cardiac and skeletal muscles, and has a positive role on cardiac functions (indicated by upward arrow), and muscular proteins in zebrafish. Knockdown of irisin played an important role in modulating muscle protein encoding mRNAs and cardiovascular physiology in zebrafish (indicated by down arrow). Together, irisin is a positive modulator of cardiac physiology and regulates muscle proteins in zebrafish.

In conclusion, this research using approaches to add or remove irisin, indicate a primarily positive role for this peptide on zebrafish cardiac function. While some of our results are in agreement with similar studies in rodents, it appears that species-specific differences in irisin actions exist. We found muscle proteins as novel targets of irisin, and this suggest a modulatory role for these proteins in regulating cardiac function. Fig 8 summarizes the effects of irisin on cardiovascular physiology and regulation of muscle proteins in zebrafish. Our results establish irisin as a potent bioactive molecule in zebrafish. It also provides the basis for future research on the mechanisms of action of irisin, and its role in other physiological processes in zebrafish.
